# Supplementary material for: FBXO32 promotes microenvironment underlying epithelial-mesenchymal transition via CtBP1 during tumour metastasis and brain development
Source: Nat Commun. 2017 Nov 15;8:1523. doi: 10.1038/s41467-017-01366-x (PMC5688138; doi:10.1038/s41467-017-01366-x)
Supplement: Supplementary file 1 — Supplementary Information [file 41467_2017_1366_MOESM1_ESM.pdf]

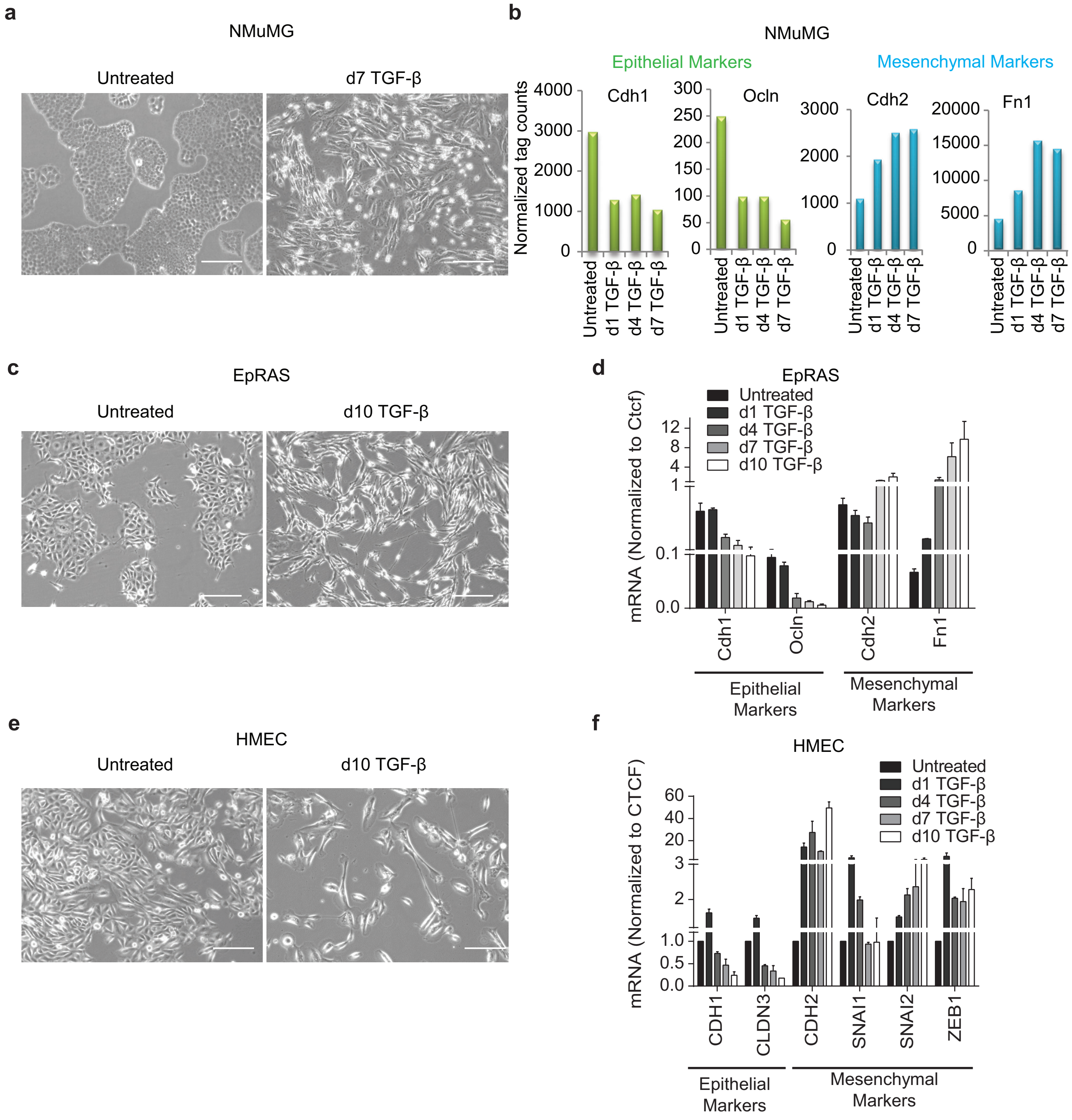
a
NMuMG
Untreated
d7 TGF-β
b
NMuMG
Epithelial Markers
Mesenchymal Markers
Normalized tag counts
Cdh1
Ocln
Cdh2
Fn1
4000
3000
2000
1000
0
300
250
200
150
100
50
0
3000
2000
1000
0
20000
15000
10000
5000
0
Untreated
d1 TGF-β
d4 TGF-β
d7 TGF-β
c
EpRAS
Untreated
d10 TGF-β
d
EpRAS
Untreated
d1 TGF-β
d4 TGF-β
d7 TGF-β
d10 TGF-β
mRNA (Normalized to Ctcf)
12
8
4
1
0.1
0.0
Cdh1
Ocln
Cdh2
Fn1
Epithelial Markers
Mesenchymal Markers
e
HMEC
Untreated
d10 TGF-β
f
HMEC
Untreated
d1 TGF-β
d4 TGF-β
d7 TGF-β
d10 TGF-β
mRNA (Normalized to CTCF)
60
40
20
3
2
1.0
0.5
0.0
CDH1
CLDN3
CDH2
SNAI1
SNAI2
ZEB1
Epithelial Markers
Mesenchymal Markers

Supplementary Figure 1. Model systems for EMT.

(Related to Fig 1)

(a) Representative bright field images of NMuMG cells undergoing TGF-β induced EMT. **(b)** RNA-seq data showing mRNA level of key EMT relevant genes in NMuMG cells undergoing EMT, the y-axis represents the normalized tag count. **(c)** Representative bright field images of mouse EpRAS cell line undergoing TGF-β induced EMT. **(d)** mRNA levels of key EMT markers during EpRAS cell line undergoing TGF-β induced EMT measured by RT-PCR relative to Ctcf and plotted on y-axis. **(e)** Representative bright field images Human Primary Mammary Epithelial Cells (HMEC) undergoing TGF-β induced EMT. **(f)** mRNA levels of key EMT markers during HMEC cell undergoing TGF-β induced EMT measured by RT-PCR relative to CTCF and plotted on y-axis. All experimental conclusions or representative images were drawn from three independent biological replicates unless otherwise specified in the text.

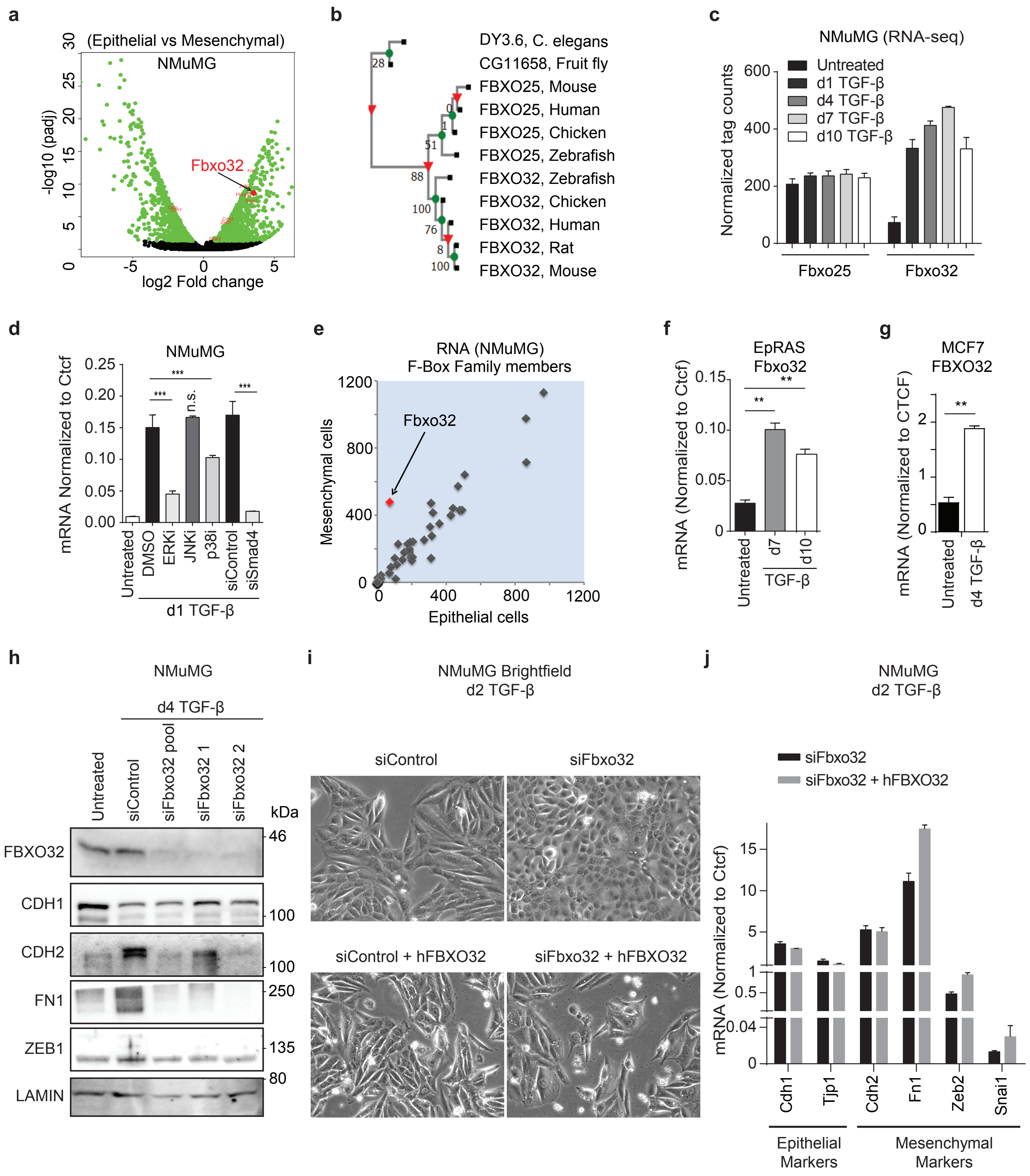
a
(Epithelial vs Mesenchymal)
NMuMG
Fbxo32
-log10 (padj)
log2 Fold change
b
DY3.6, C. elegans
CG11658, Fruit fly
FBXO25, Mouse
FBXO25, Human
FBXO25, Chicken
FBXO25, Zebrafish
FBXO32, Zebrafish
FBXO32, Chicken
FBXO32, Human
FBXO32, Rat
FBXO32, Mouse
c
NMuMG (RNA-seq)
Untreated
d1 TGF-β
d4 TGF-β
d7 TGF-β
d10 TGF-β
Normalized tag counts
Fbxo25
Fbxo32
d
NMuMG
mRNA Normalized to Ctcf
Untreated
DMSO
ERKi
JNKi
p38i
siControl
siSmad4
d1 TGF-β
n.s.
e
RNA (NMuMG)
F-Box Family members
Fbxo32
Mesenchymal cells
Epithelial cells
f
EpRAS
Fbxo32
mRNA (Normalized to Ctcf)
Untreated
d7
d10
TGF-β
g
MCF7
FBXO32
mRNA (Normalized to CTCF)
Untreated
d4 TGF-β
h
NMuMG
d4 TGF-β
Untreated
siControl
siFbxo32 pool
siFbxo32 1
siFbxo32 2
kDa
FBXO32
CDH1
CDH2
FN1
ZEB1
LAMIN
i
NMuMG Brightfield
d2 TGF-β
siControl
siFbxo32
siControl + hFBXO32
siFbxo32 + hFBXO32
j
NMuMG
d2 TGF-β
siFbxo32
siFbxo32 + hFBXO32
mRNA (Normalized to Ctcf)
Cdh1
Tjp1
Cdh2
Fn1
Zeb2
Snai1
Epithelial Markers
Mesenchymal Markers

Supplementary Figure 2. Fbxo32 is induced during EMT.

(Related to Fig 1-2)

(a) Volcano plot showing significantly differentially expressed genes in both epithelial and mesenchymal NMuMG cells. **(b)** Cladogram based on alignment of all homologous sequences (protein domain) in the TreeFam family showing relationships between Fbxo32 genes in different species. Few models species were shown for simplicity reasons. Numbers below branches are bootstrap values, whereby 100% indicates strong support for the node, and lower values represent weaker node. Cladogram was created by using the resource TreeFam, freely available at http://www.treefam.org/family. **(c)** RNA-seq data showing mRNA level of Fbxo32 and Fbxo25 during TGF-β induced EMT, y-axis represents normalized tag count. **(d)** Using RT PCR mRNA levels of Fbxo32 measured relative to CTCF in NMuMG cells undergoing TGF-β induced EMT and treated with inhibitors of various Map Kinases or depleted for Smad4. **(e)** Comparison of the differential expression, quantified as normalized tag counts, of F-box family members in epithelial and mesenchymal NMuMG cells. **(f)** mRNA level for Fbxo32 during TGF-β induced EMT in EpRAS were measured by RT-PCRs relative to Ctcf and plotted on y-axis. **(g)** mRNA level for FBXO32 during TGF-β induced EMT in MCF7 were measured by RT-PCRs relative to CTCF and plotted on y-axis. **(h)** Western blot analysis of FBXO32 along with key EMT markers Cdh1, Cdh2, FN1, and Zeb1 in NMuMG cells undergoing TGF-β-induced EMT and depleted for Fbxo32, either with pool of siRNAs or single siRNA. Lamin acted as a loading control. **(i)** Representative bright-field images showing the morphology of NMuMG cell undergoing TGF-β-induced EMT and upon siRNA mediated depletion of Fbxo32 and rescue by overexpression of siRNA resistant human FBXO32 for two days. **(j)** Using RT-PCR, mRNA levels of key EMT markers in in above condition as mentioned in (i) were measured relative to Ctcf and plotted on the y-axis. Error bars represent the SEM of three independent biological replicates. *p<0.05, **p<0.01, ***p<0.001, Student's t-test.

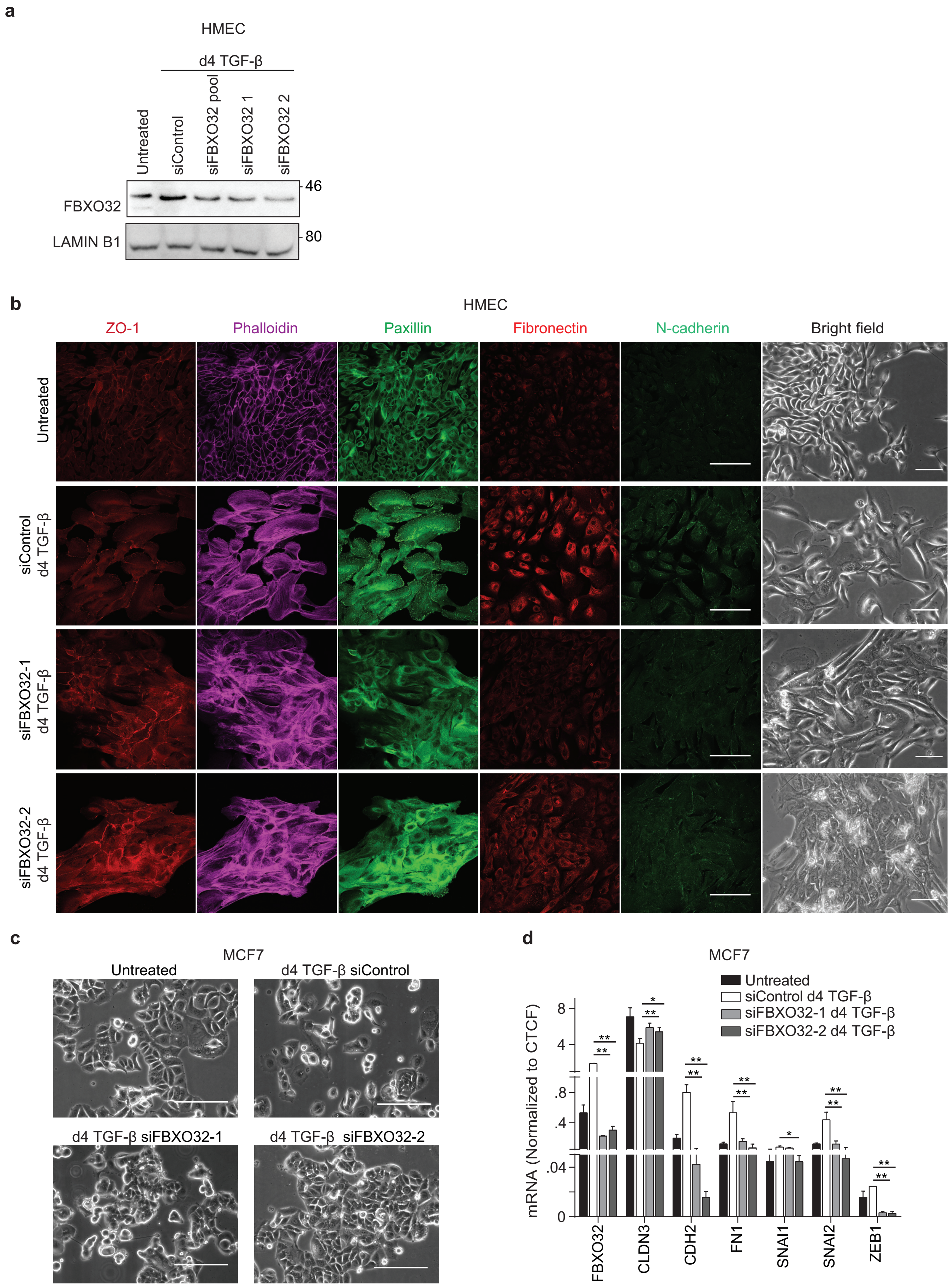
a
HMEC
d4 TGF-β
Untreated
siControl
siFBXO32 pool
siFBXO32 1
siFBXO32 2
FBXO32
46
LAMIN B1
80
b
HMEC
ZO-1
Phalloidin
Paxillin
Fibronectin
N-cadherin
Bright field
Untreated
siControl d4 TGF-β
siFBXO32-1 d4 TGF-β
siFBXO32-2 d4 TGF-β
c
MCF7
Untreated
d4 TGF-β siControl
d4 TGF-β siFBXO32-1
d4 TGF-β siFBXO32-2
d
MCF7
Untreated
siControl d4 TGF-β
siFBXO32-1 d4 TGF-β
siFBXO32-2 d4 TGF-β
mRNA (Normalized to CTCF)
8
4
.8
.4
.04
0
FBXO32
CLDN3
CDH2
FN1
SNAI1
SNAI2
ZEB1

Supplementary Figure 3. FBXO32 is critical for human EMT.

(Related to Figure 1-2)

(a) Western blot analysis of FBXO32 in HMEC cells undergoing TGF-β-induced EMT and depleted for FBXO32, either with pool of siRNAs or single siRNA. Lamin acted as a loading control. **(b)** Representative bright-field and immunofluorescence images showing the localization and expression levels of EMT marker proteins after four days (d4) of independent siRNAs-mediated depletion of FBXO32 compared to non-targeting control (siControl) in HMEC. Staining was performed to assess the expression with antibodies against the epithelial marker ZO1, the mesenchymal marker N-cadherin, Fibronectin-1, Phalloidin (to visualize the actin cytoskeleton) and Paxillin (to detect focal adhesion plaques). Scale bar, 100 μm. **(c)** Representative bright-field images showing the morphology after four days (d4) of independent siRNAs-mediated depletion of FBXO32, compared to siControl in MCF7 undergoing TGF- β-induced EMT. **(d)** Using RT-PCR, mRNA levels of key EMT markers in MCF7 cells transfected with either control siRNA or independent siRNAs against FBXO32 during TGF-β-induced EMT were measured relative to CTCF and plotted on the y-axis. Error bars represent the SEM of three independent biological replicates. *p<0.05, **p<0.01, ***p<0.001, Student's t-test.

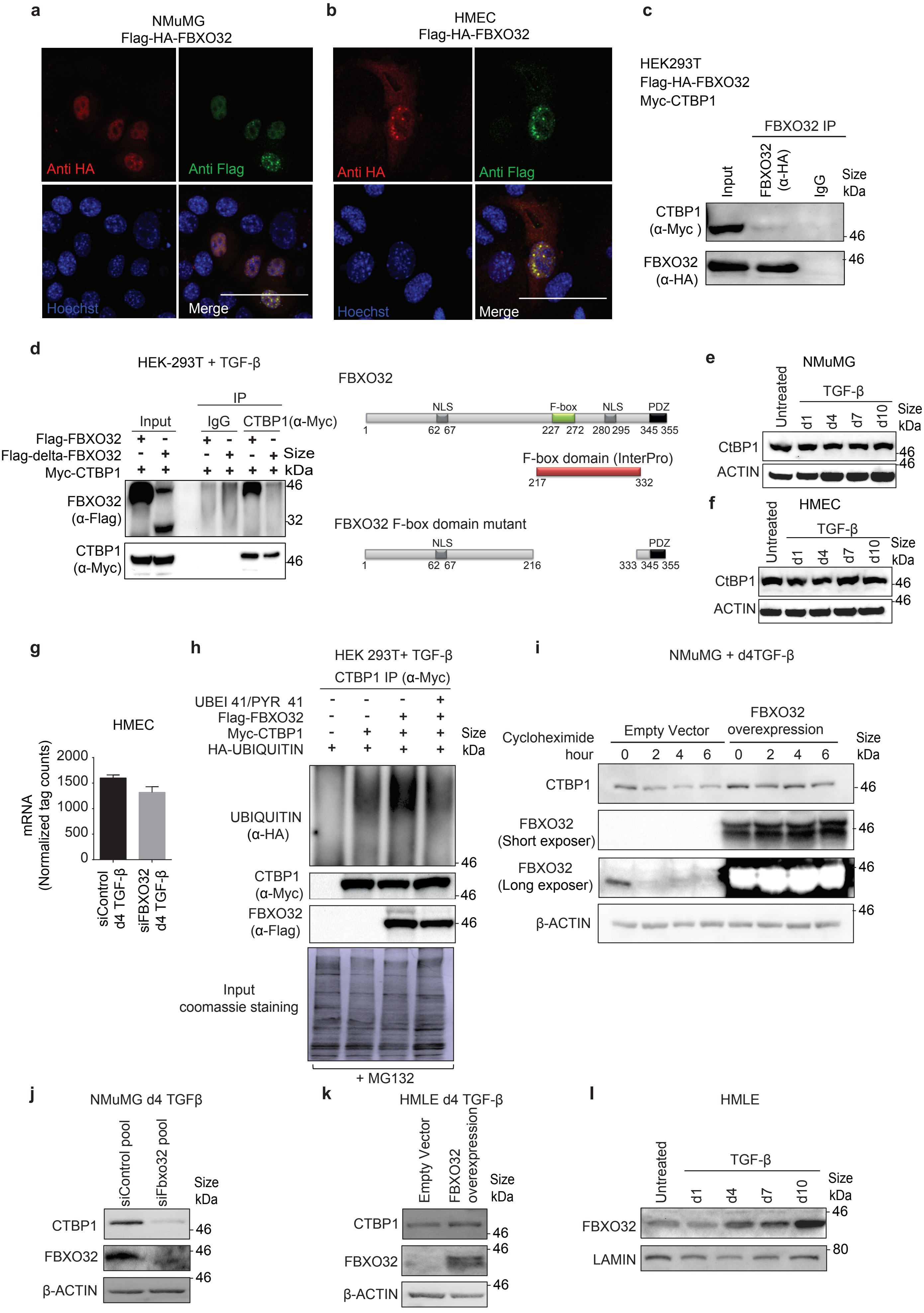
a
NMuMG
Flag-HA-FBXO32
Anti HA
Anti Flag
Hoechst
Merge
b
HMEC
Flag-HA-FBXO32
Anti HA
Anti Flag
Hoechst
Merge
c
HEK293T
Flag-HA-FBXO32
Myc-CTBP1
FBXO32 IP
Input
FBXO32 (α-HA)
IgG
Size kDa
CTBP1 (α-Myc)
FBXO32 (α-HA)
46
46
d
HEK-293T + TGF-β
IP
Input
IgG
CTBP1(α-Myc)
Flag-FBXO32
Flag-delta-FBXO32
Myc-CTBP1
Size kDa
FBXO32 (α-Flag)
CTBP1 (α-Myc)
46
32
46
FBXO32
NLS
F-box
NLS
PDZ
1
62 67
227 272
280 295
345 355
F-box domain (InterPro)
217
332
FBXO32 F-box domain mutant
NLS
PDZ
1
62 67
216
333 345 355
e
NMuMG
Untreated
TGF-β
d1
d4
d7
d10
Size kDa
CtBP1
ACTIN
46
46
f
HMEC
Untreated
TGF-β
d1
d4
d7
d10
Size kDa
CtBP1
ACTIN
46
46
g
HMEC
mRNA (Normalized tag counts)
2000
1500
1000
500
0
siControl d4 TGF-β
siFBXO32 d4 TGF-β
h
HEK 293T+ TGF-β
CTBP1 IP (α-Myc)
UBEI 41/PYR 41
Flag-FBXO32
Myc-CTBP1
HA-UBIQUITIN
Size kDa
UBIQUITIN (α-HA)
CTBP1 (α-Myc)
FBXO32 (α-Flag)
Input coomassie staining
46
46
46
+ MG132
i
NMuMG + d4TGF-β
Cycloheximide hour
Empty Vector
FBXO32 overexpression
0 2 4 6
0 2 4 6
Size kDa
CTBP1
FBXO32 (Short exposer)
FBXO32 (Long exposer)
β-ACTIN
46
46
46
46
j
NMuMG d4 TGFβ
siControl pool
siFbxo32 pool
Size kDa
CTBP1
FBXO32
β-ACTIN
46
46
46
k
HMLE d4 TGF-β
Empty Vector
FBXO32 overexpression
Size kDa
CTBP1
FBXO32
β-ACTIN
46
46
46
l
HMLE
Untreated
TGF-β
d1
d4
d7
d10
Size kDa
FBXO32
LAMIN
46
80

Supplementary Figure 4. FBXO32 mediates stability of CTBP1.

(Related to Fig 3)

(a-b) immunofluorescence images showing localization of FBXO32 in NMuMG (a) and HMEC (b) cells transfected with Flag-HA-FBXO32. **(c)** Western blot of immunoprecipitated samples to validate FBXO32 and CTBP1 interactions. HEK293T cells were co-transfected with Myc-CTBP1 and Flag-HA-FBXO32 and anti-FBXO32 IP was performed. Immunoblot was performed to detect CTBP1 and FBXO32. **(d)** Western blot of immunoprecipitated samples to validate FBXO32 and CTBP1 interactions. HEK293T cells were co-transfected with Myc-CTBP1 and Flag-FBXO32 full length construct or Flag-FBXO32 F-box domain deleted construct. CTBP1 IP was performed with anti Myc antibody. Immunoblot was performed to detect FBXO32 and CTBP1. **(e-f)** Western blot analysis of CTBP1 in mouse NMuMG cell (e) and human HMECs (f) undergoing TGF-β-induced EMT. β-Actin acted as a loading control. **(g)** RNA-seq data showing mRNA level of CTBP1 in HMEC cell undergoing TGF-β induced EMT for 4 days, y-axis represents normalized tag count. **(h)** HEK293 cells were transfected with various tagged construct as mentioned in the figure. Four hours before immunoprecipitation MG132 was added in all conditions. Ubiquitination pathway blocker UBEI41/ PYR 41 was also added four hours before IP as mentioned in the figure. Western blot for CTBP1 (α-Myc) immunoprecipitated samples was performed to detect ubiquitin (α-HA) and CTBP1 and FBXO32. **(i)** Western blot showing CTBP1 protein levels at various time points after cyclohexamide treatment in NMuMG cells stably expressing empty vector or FBXO32 overexpression construct. β-Actin acted as a loading control **(j)** Western blot showing CTBP1 protein stability in NMuMG cells transfected with control siRNA or siRNA against Fbxo32. β-ACTIN act as loading control. **(k)** Western blot showing CTBP1 protein levels in HMLE cells transfected with either empty vector or FBXO32 overexpression construct or control siRNA or siRNA against FBXO32. β-ACTIN act as loading control. **(l)** Western blot analysis of FBXO32 in human HMLE cells undergoing TGF-β-induced EMT. LAMIN-B1 acted as a loading control.

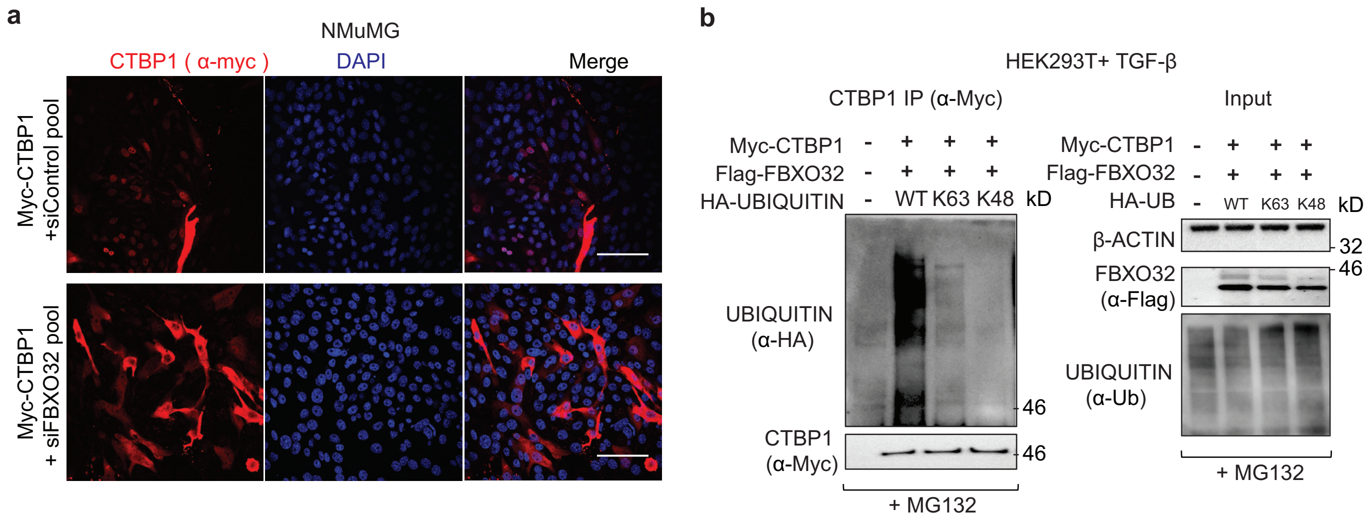

a
NMuMG
CTBP1 (α-myc)
DAPI
Merge
Myc-CTBP1 +siControl pool
Myc-CTBP1 + siFBXO32 pool
b
HEK293T+ TGF-β
CTBP1 IP (α-Myc)
Input
Myc-CTBP1 - + + +
Flag-FBXO32 - + + +
HA-UBIQUITIN - WT K63 K48 kD
UBIQUITIN (α-HA)
46
CTBP1 (α-Myc)
46
+ MG132
Myc-CTBP1 - + + +
Flag-FBXO32 - + + +
HA-UB - WT K63 K48 kD
β-ACTIN
32
FBXO32 (α-Flag)
46
UBIQUITIN (α-Ub)
+ MG132

Supplementary Figure 5. FBXO32 mediated K63 ubiquitination leads to nuclear retention of CTBP1.

(Related to Figure 3)

(a) Immunofluorescence image showing CTBP1 localization in control and Fbxo32-depleted NMuMG cells undergoing EMT. Scale bar, 100 μm. **(b)** A similar analysis as in (Figure 3g), but co-transfected with modified ubiquitin to demonstrate FBXO32-mediated K63-linked ubiquitination of CTBP1. Here, K63 represents ubiquitin with all of the lysines modified to arginines, except the lysine at position 63. Similarly, in K48, all of the lysines were modified to arginines, except the lysine at position 48.

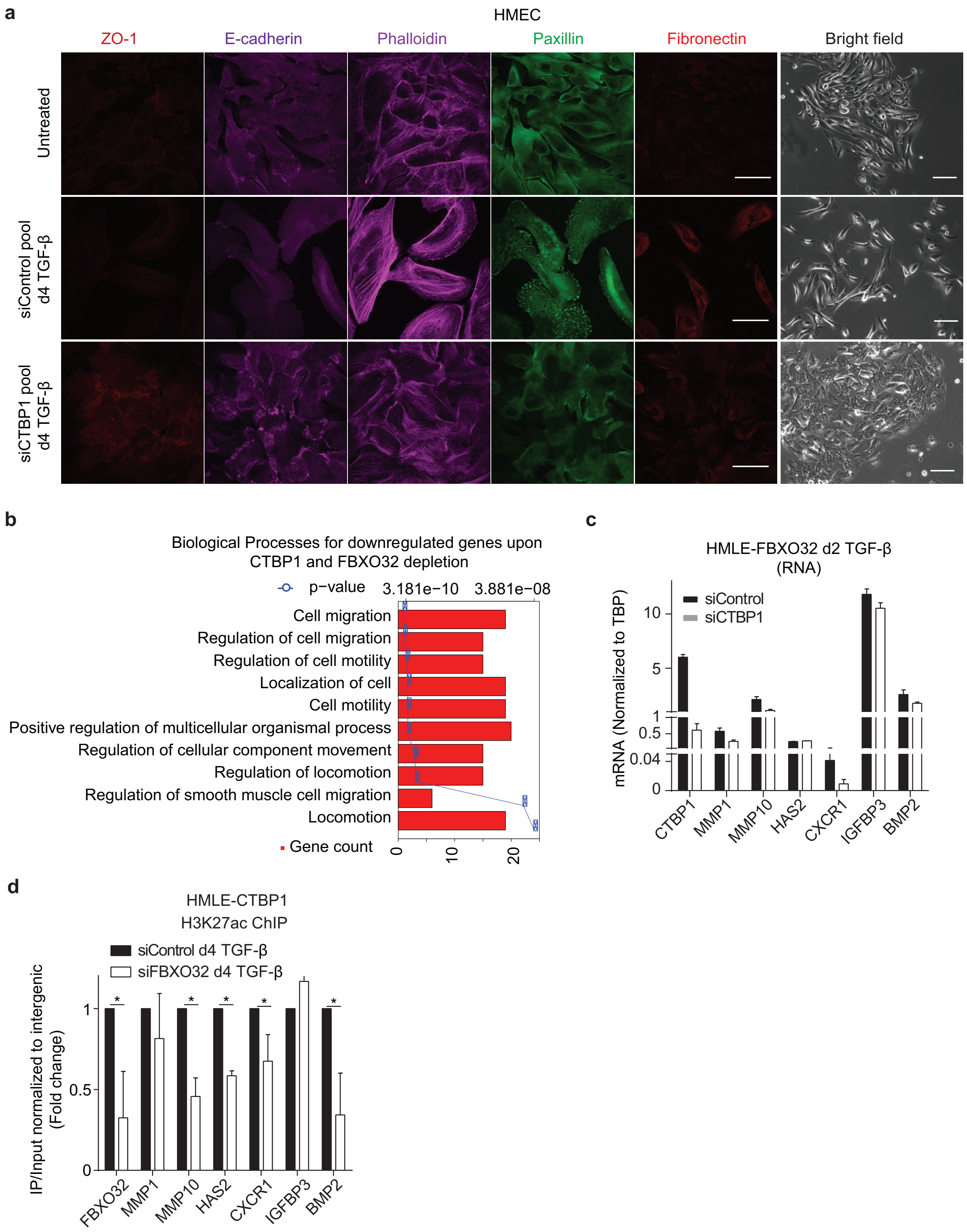
a
HMEC
ZO-1
E-cadherin
Phalloidin
Paxillin
Fibronectin
Bright field
Untreated
siControl pool d4 TGF-β
siCTBP1 pool d4 TGF-β
b
Biological Processes for downregulated genes upon CTBP1 and FBXO32 depletion
p−value 3.181e−10 3.881e−08
Cell migration
Regulation of cell migration
Regulation of cell motility
Localization of cell
Cell motility
Positive regulation of multicellular organismal process
Regulation of cellular component movement
Regulation of locomotion
Regulation of smooth muscle cell migration
Locomotion
Gene count
0
10
20
c
HMLE-FBXO32 d2 TGF-β (RNA)
siControl
siCTBP1
mRNA (Normalized to TBP)
10
5
1
0.5
0.04
0
CTBP1
MMP1
MMP10
HAS2
CXCR1
IGFBP3
BMP2
d
HMLE-CTBP1
H3K27ac ChIP
siControl d4 TGF-β
siFBXO32 d4 TGF-β
IP/Input normalized to intergenic (Fold change)
1
0.5
0
FBXO32
MMP1
MMP10
HAS2
CXCR1
IGFBP3
BMP2

Supplementary Figure 6. CTBP1 depletion results in a reversal of EMT in human primary epithelial cells.

(Related to Fig 4)

(a) Representative bright field and immunofluorescence images for localization and expression levels of EMT marker proteins after four days of siRNA-mediated depletion of CTBP1 compared to control siRNA in HMEC. Staining was performed with antibodies against epithelial markers ZO1 and E-cadherin, mesenchymal markers Fibronectin-1, with Phalloidin to visualize the actin cytoskeleton and against Paxillin to detect focal adhesion plaques. Scale bar, 100 µm. **(b)** GO analysis of genes downregulated upon CTBP1 and FBXO32 knockdown in HMEC undergoing EMT. **(c)** Using RT-PCR, the levels of FBXO32 and CTBP1 target genes in HMLE cells undergoing TGF-β-induced EMT for two days (d2) and transfected with either control siRNA or siRNAs against CTBP1 in FBXO32 overexpression background were measured relative to CTCF and plotted on the y-axis. **(d)** ChIP assay using anti-H3K27ac antibody following expression of Myc-CTBP1 in HMLE cells induced by TGF-β for 4 days and transfected with control siRNA or siRNA against FBXO32. Quantitative PCRs were performed for indicating gene promoters and enrichments are plotted on the y-axis as the ratio of precipitated DNA (bound) to total input DNA and normalized to intergenic region (in fold change to control siRNA). SEM is derived from independent biological replicates. For all above experiments the error bars represent the SEM of three independent biological replicates. *$p<0.05$, **$p<0.01$, ***$p<0.001$, Student's t-test.

a

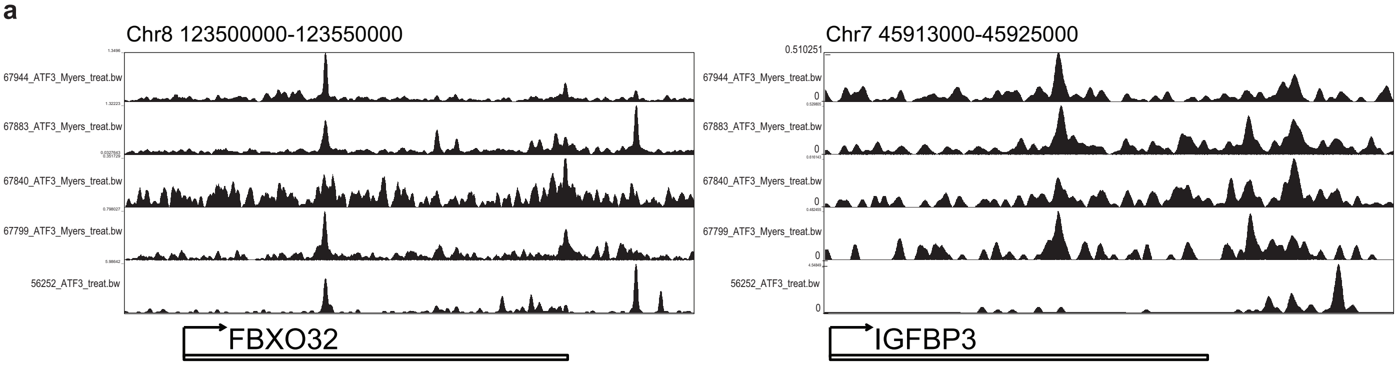
Chr8 123500000-123550000
67944_ATF3_Myers_treat.bw
67883_ATF3_Myers_treat.bw
67840_ATF3_Myers_treat.bw
67799_ATF3_Myers_treat.bw
56252_ATF3_treat.bw
FBXO32
Chr7 45913000-45925000
0.510251
0
IGFBP3

b

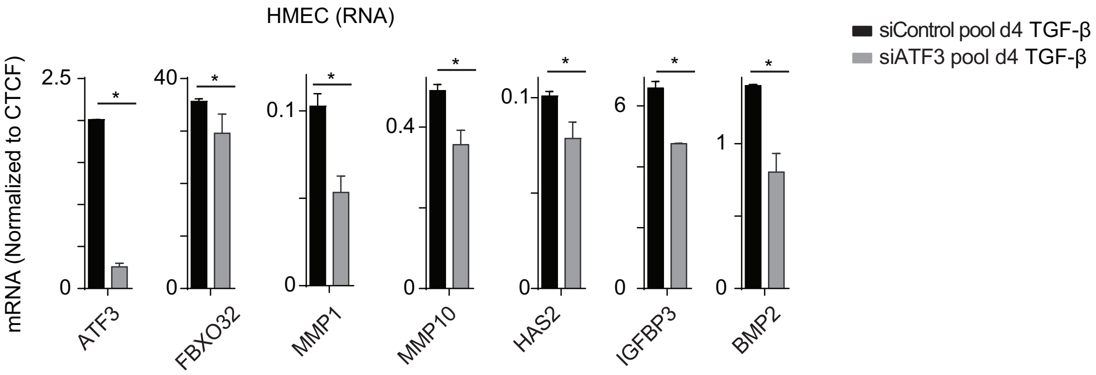
HMEC (RNA)
siControl pool d4 TGF-β
siATF3 pool d4 TGF-β
mRNA (Normalized to CTCF)
2.5
40
0.1
0.4
0.1
6
1
0
*
ATF3
FBXO32
MMP1
MMP10
HAS2
IGFBP3
BMP2

Supplementary Figure 7. FBXO32 mediates transcriptional alterations via distinct transcription factors.

(Related to Fig 4)

(a) Genome browser tracks derived from a publicly available ATF3 ChIP-seq dataset (ENCODE/HAIB) showing binding of ATF3 at promoters and/or distal regions of genes downregulated upon FBXO32 and CTBP1 knockdown during EMT in human primary epithelial cells. **(b)** mRNA levels of key EMT-associated deregulated genes upon ATF3 depletion for 4 days in HMECs undergoing TGF-β-induced EMT were measured relative to CTCF via RT-PCR, and the results were plotted on the y-axis. Error bars represent the SEM of three independent biological replicates. *p<0.05, Student's t-test.

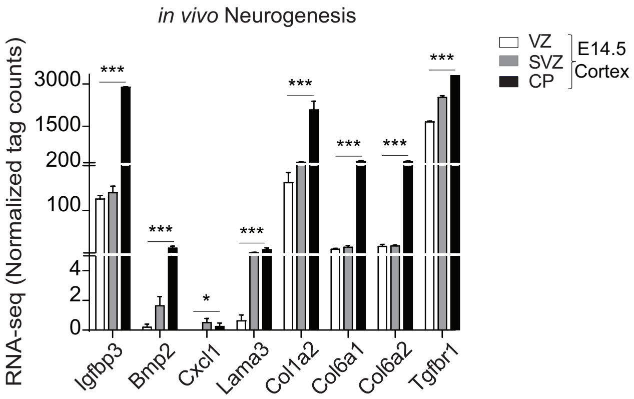

in vivo Neurogenesis
RNA-seq (Normalized tag counts)
3000
1500
200
100
4
2
0
***
***
*
***
***
***
***
***
Igfbp3
Bmp2
Cxcl1
Lama3
Col1a2
Col6a1
Col6a2
Tgfbr1
VZ
SVZ
CP
E14.5
Cortex

Supplementary Figure 8. FBXO32 is required for neuronal EMT.

(Related to Fig 5)

Normalized tag counts derived from the *in vivo* neurogenesis RNA-seq data used in Figure 5b, showing the mRNA levels of genes that were downregulated in Fbxo32-ablated HMEC. The error bars represent the SEM of three independent biological replicates. *$p<0.05$, **$p<0.01$, ***$p<0.001$, Student's t-test.

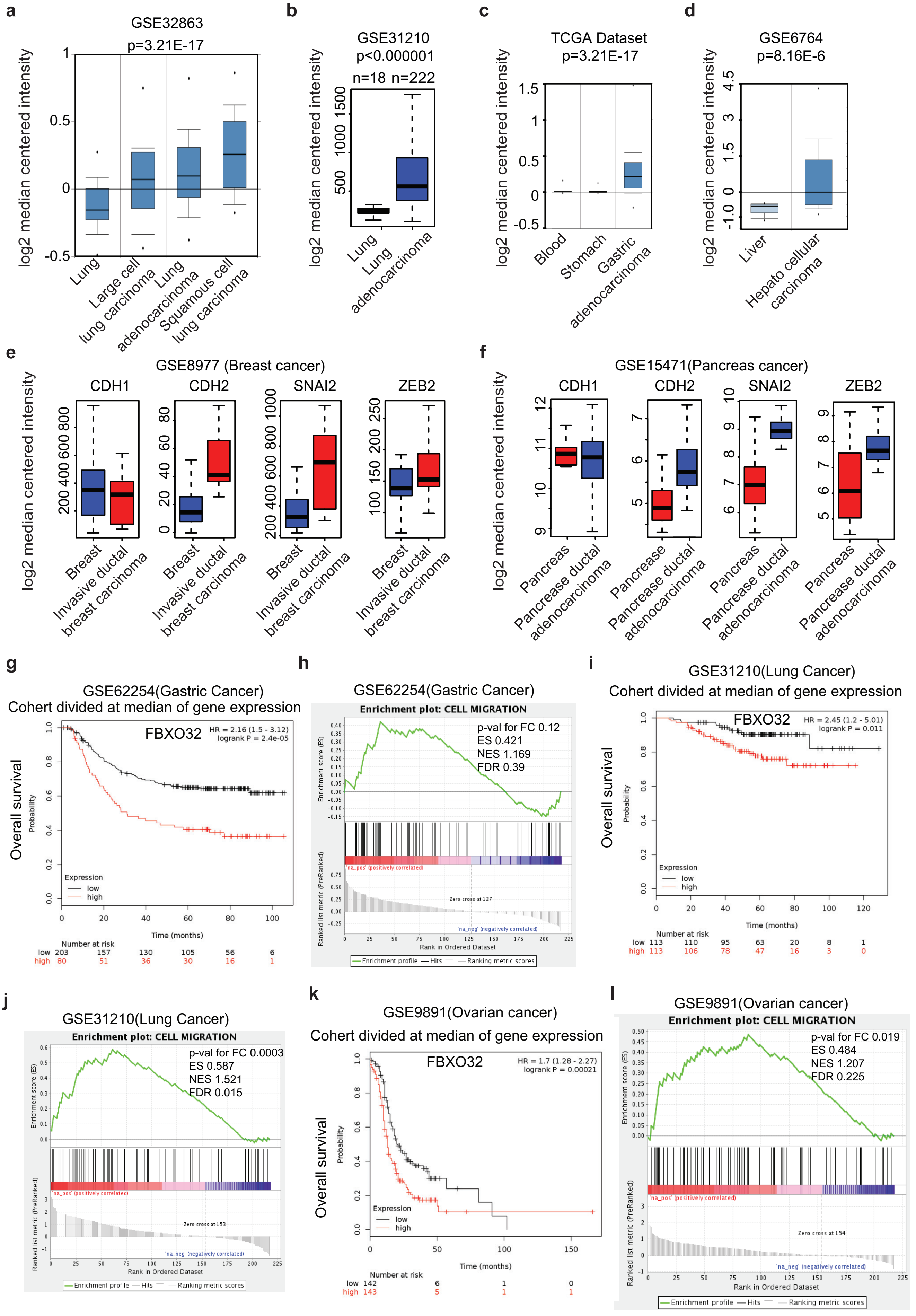

a
GSE32863
p=3.21E-17
log2 median centered intensity
Lung
Large cell lung carcinoma
Lung adenocarcinoma
Squamous cell lung carcinoma
b
GSE31210
p<0.000001
n=18 n=222
Lung
Lung adenocarcinoma
c
TCGA Dataset
p=3.21E-17
Blood
Stomach
Gastric adenocarcinoma
d
GSE6764
p=8.16E-6
Liver
Hepato cellular carcinoma
e
GSE8977 (Breast cancer)
CDH1
CDH2
SNAI2
ZEB2
Breast
Invasive ductal breast carcinoma
f
GSE15471(Pancreas cancer)
Pancreas
Pancrease ductal adenocarcinoma
g
GSE62254(Gastric Cancer)
Cohort divided at median of gene expression
FBXO32
HR = 2.16 (1.5 - 3.12)
logrank P = 2.4e-05
Overall survival
Probability
Expression
low
high
Time (months)
Number at risk
low 203 157 130 105 56 6
high 80 51 36 30 16 1
h
GSE62254(Gastric Cancer)
Enrichment plot: CELL MIGRATION
p-val for FC 0.12
ES 0.421
NES 1.169
FDR 0.39
Enrichment score (ES)
Ranked list metric (PreRanked)
'na_pos' (positively correlated)
Zero cross at 127
'na_neg' (negatively correlated)
Rank in Ordered Dataset
Enrichment profile — Hits — Ranking metric scores
i
GSE31210(Lung Cancer)
Cohort divided at median of gene expression
FBXO32
HR = 2.45 (1.2 - 5.01)
logrank P = 0.011
Overall survival
Probability
Expression
low
high
Time (months)
Number at risk
low 113 110 95 63 20 8 1
high 113 106 78 47 16 3 0
j
GSE31210(Lung Cancer)
Enrichment plot: CELL MIGRATION
p-val for FC 0.0003
ES 0.587
NES 1.521
FDR 0.015
'na_pos' (positively correlated)
Zero cross at 153
'na_neg' (negatively correlated)
Rank in Ordered Dataset
Enrichment profile — Hits — Ranking metric scores
k
GSE9891(Ovarian cancer)
Cohort divided at median of gene expression
FBXO32
HR = 1.7 (1.28 - 2.27)
logrank P = 0.00021
Overall survival
Probability
Expression
low
high
Time (months)
Number at risk
low 142 6 1 0
high 143 5 1 1
l
GSE9891(Ovarian cancer)
Enrichment plot: CELL MIGRATION
p-val for FC 0.019
ES 0.484
NES 1.207
FDR 0.225
'na_pos' (positively correlated)
Zero cross at 154
'na_neg' (negatively correlated)
Rank in Ordered Dataset
Enrichment profile — Hits — Ranking metric scores

Supplementary Figure 9. FBXO32 expression correlates with tumor aggressiveness.

(Related to Fig 6)

(a-d) Box plots showing level of FBXO32 expression in normal vs tumor samples in various well characterized clinical tumor databases, in Lung (a-b), in Stomach (c), and in liver (d). **(e-f)** Box plots showing level of key EMT markers in normal vs tumor condition in breast tumor datasets used in Fig 5b-c respectively. Box plots in Supplementary Figure 9a-f, represent the 25th to 75th quartiles with the bold horizontal line representing the median value. **(g)** A Kaplan-Meier analysis was performed for the Colon tumor dataset and survival curves showing decreased metastasis free survival, which was significantly correlated with higher FBXO32 expression. **(h)** A gene set enrichment analysis shows correlation between genes downregulated upon FBXO32 depletion and the genes associated to the specified GO terms as a function of fold change between expression levels of these genes in high FBXO32 and low FBXO32 expression in characterized breast tumor datasets used in Fig S7g. **(i-j)** Similar analysis as in Fig S7g-h, but in lung carcinoma dataset. **(k-l)** Similar analysis as in Fig S7g and h, but in ovarian carcinoma dataset. P value for fold change, Enrichment score (ES), Normalized Enrichment Score (NES) and FDR q-value were provided in the figure for all gene set enrichment analysis.

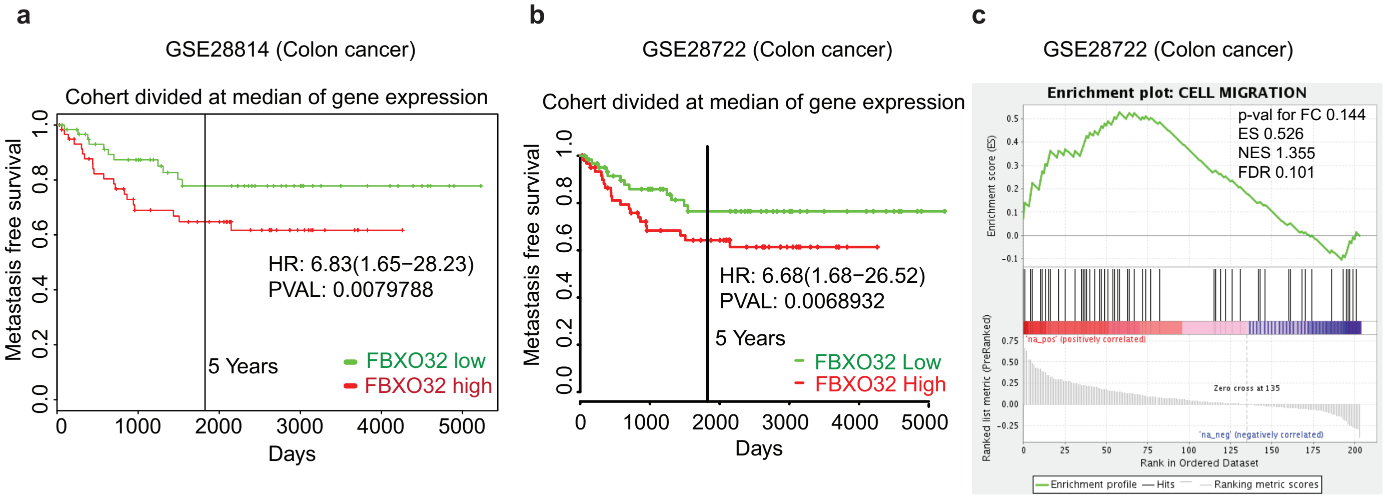
a
GSE28814 (Colon cancer)
Cohert divided at median of gene expression
Metastasis free survival
Days
HR: 6.83(1.65−28.23)
PVAL: 0.0079788
5 Years
FBXO32 low
FBXO32 high
b
GSE28722 (Colon cancer)
Cohert divided at median of gene expression
Metastasis free survival
Days
HR: 6.68(1.68−26.52)
PVAL: 0.0068932
5 Years
FBXO32 Low
FBXO32 High
c
GSE28722 (Colon cancer)
Enrichment plot: CELL MIGRATION
p-val for FC 0.144
ES 0.526
NES 1.355
FDR 0.101
Enrichment score (ES)
'na_pos' (positively correlated)
Zero cross at 135
'na_neg' (negatively correlated)
Ranked list metric (PreRanked)
Rank in Ordered Dataset
Enrichment profile
Hits
Ranking metric scores

Supplementary Figure 10. FBXO32 expression correlates with tumor aggressiveness.

(Related to Fig 6)

(a) A Kaplan-Meier analysis was performed for the Colon tumor dataset GSE28814 and survival curves showing decreased metastasis free survival which was significantly correlated with higher FBXO32 expression. **(b)** Similar analysis as in Fig S8a but in another Colon tumor dataset GSE28722. **(c)** Gene set enrichment analysis showing correlation between genes downregulated upon FBXO32 depletion and the genes associated to the specified GO terms as a function of fold change between expression levels of these genes in tumor with high FBXO32 and low FBXO32 expression in the characterized tumor datasets in Fig S8b. p-value for fold change, Enrichment score (ES), Normalized Enrichment Score (NES) and FDR q-value were provided in the figure.

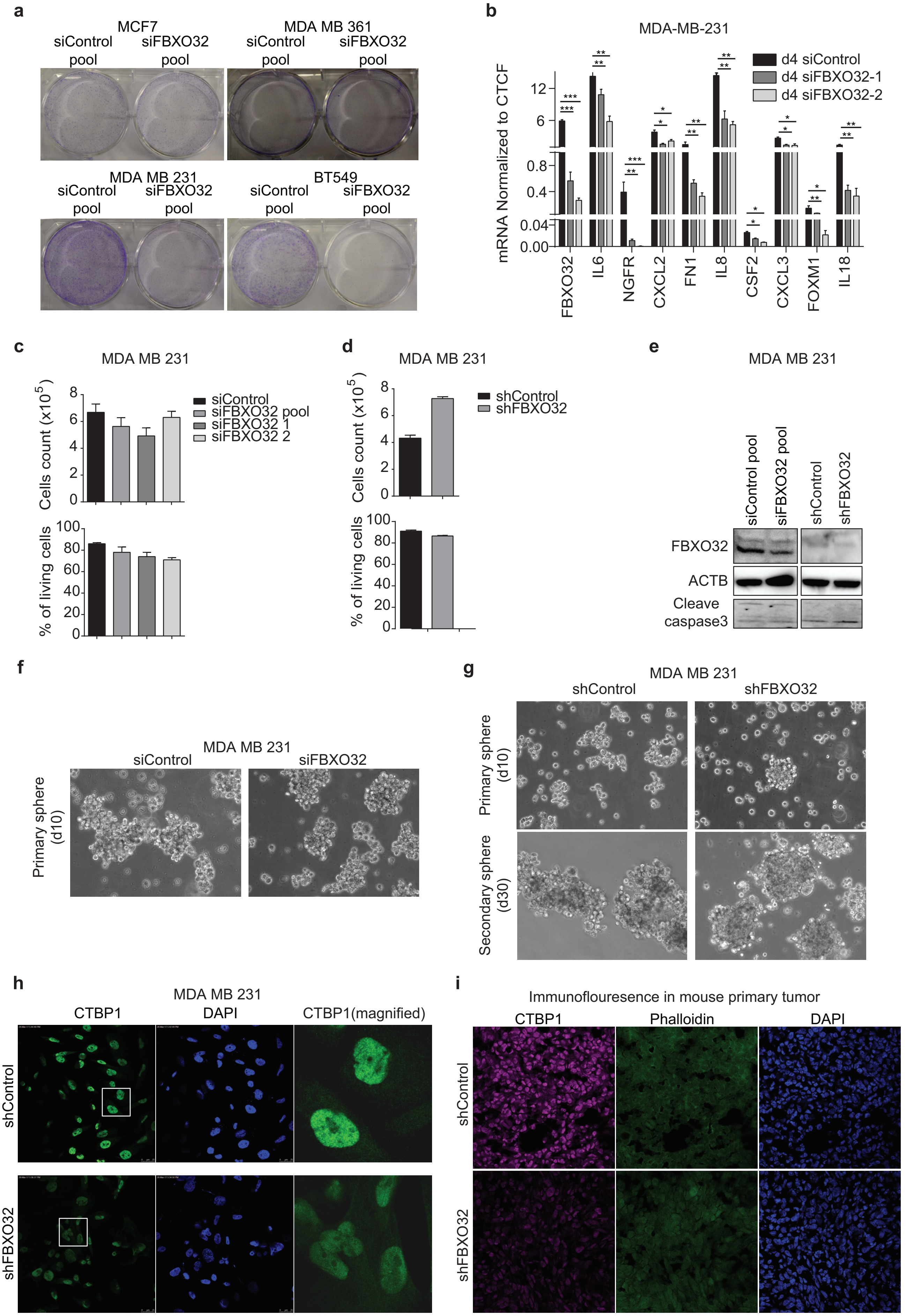
a
MCF7
siControl pool
siFBXO32 pool
MDA MB 361
siControl pool
siFBXO32 pool
MDA MB 231
siControl pool
siFBXO32 pool
BT549
siControl pool
siFBXO32 pool
b
MDA-MB-231
d4 siControl
d4 siFBXO32-1
d4 siFBXO32-2
mRNA Normalized to CTCF
12
6
0.8
0.4
0.04
0.00
FBXO32
IL6
NGFR
CXCL2
FN1
IL8
CSF2
CXCL3
FOXM1
IL18
c
MDA MB 231
Cells count (x10^5)
% of living cells
siControl
siFBXO32 pool
siFBXO32 1
siFBXO32 2
d
MDA MB 231
Cells count (x10^5)
% of living cells
shControl
shFBXO32
e
MDA MB 231
siControl pool
siFBXO32 pool
shControl
shFBXO32
FBXO32
ACTB
Cleave caspase3
f
MDA MB 231
siControl
siFBXO32
Primary sphere (d10)
g
MDA MB 231
shControl
shFBXO32
Primary sphere (d10)
Secondary sphere (d30)
h
MDA MB 231
CTBP1
DAPI
CTBP1(magnified)
shControl
shFBXO32
i
Immunoflouresence in mouse primary tumor
CTBP1
Phalloidin
DAPI
shControl
shFBXO32

Supplementary Figure 11. FBXO32 is crucial for the maintenance of mesenchymal identity in breast cancer cells.

(Related to Fig 7)

(a) Colony forming assay in two epithelial cell lines (MDA-MB-361 and MCF7) and two mesenchymal cell lines (MDA-MB-231 and BT549) showing colony formation ability in control and FBXO32 knock down conditions. **(b)** Using RT PCR mRNA levels of FBXO32 and key downregulated genes in control and FBXO32 knock down MDA-MB-231 cells for 4 days with independent siRNA were measured relative to CTCF and plotted on the y-axis. **(c-d)** Total number of cells and percentage of living cell were calculated for MDA-MB-231 upon depletion of FBXO32 by siRNA (c) and shRNA (d). **(e)** Western blot analysis of FBXO32 along with apoptotic marker Cleaved Caspase-3 in MDA-MB-231 cells depleted for FBXO32, either with pool of siRNAs or shRNA. B-Actin acted as a loading control. **(f-g)** Bright field images of MDA-MB-231 cells depleted for FBXO32 by siRNA (f) or shRNA (g), showing primary and secondary mammosphere, formed by sphere forming assay in presence of methylcellulose and absence of serum. **(h)** Immunofluorescence image in MDA-MB-231 cells showing CTBP1 localization in stably expressing shControl or shFBXO32 construct. Magnified images of the nuclei are provided in the right. **(i)** Immunofluorescence images for CTBP1 were measured in primary tumor obtained from the mouse injected with MDA-MB-231 cell containing shControl or shFBXO32 construct. Error bars represent the SEM of three independent biological replicates. *$p<0.05$, **$p<0.01$, ***$p<0.001$, Student's t-test.

Figure 1d

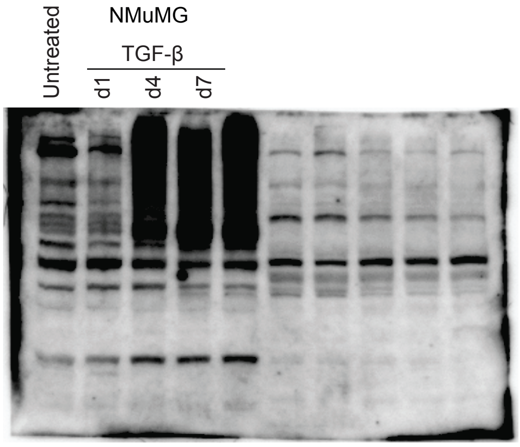

FBXO32

Figure 1e

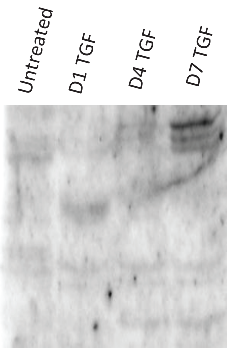

FBXO32

Figure 2d

MMPs array

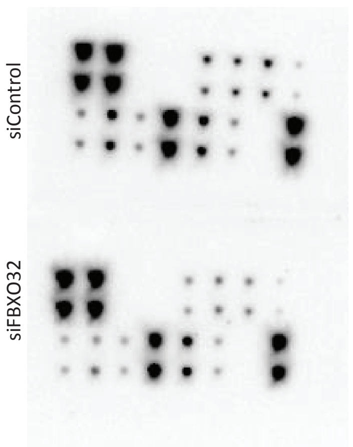

Figure 2c

Cytokine array

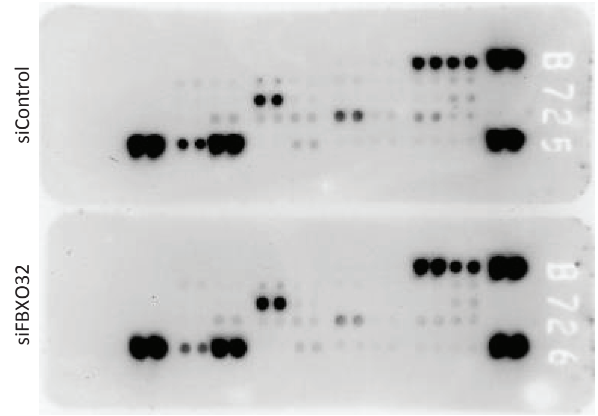

Figure 3b

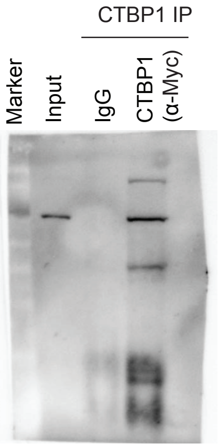

FBXO32 (α-HA)

Figure 3b

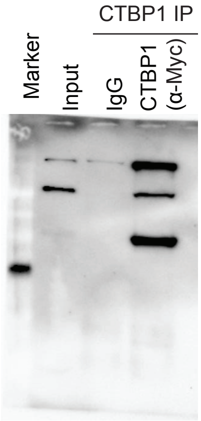

CTBP1 (α-Myc)

Figure 3c

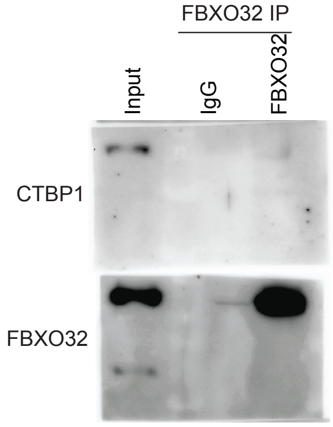

Supplementay Fig. 4c

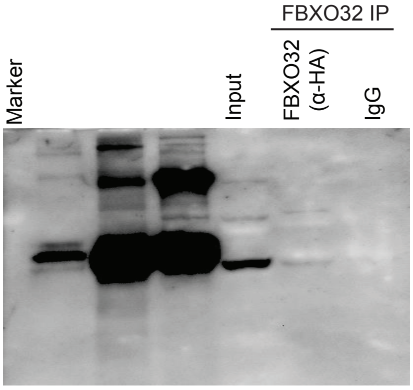

CTBP1 (α-Myc)

Figure 3d, Supplementary figure S5b

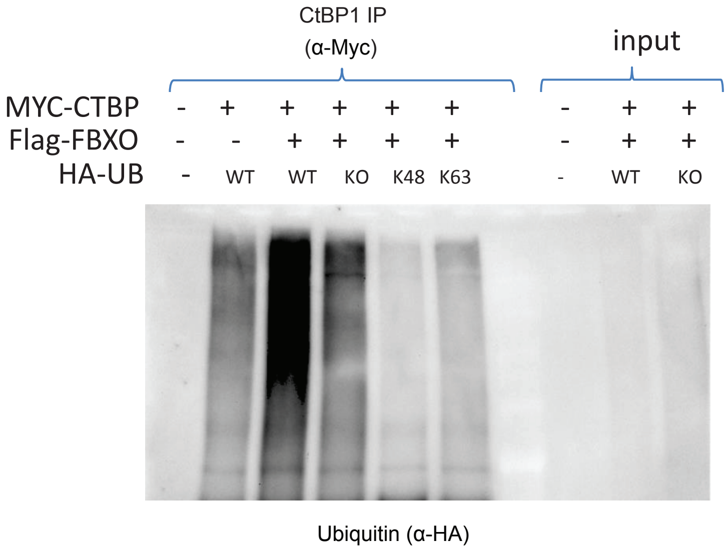

Figure 3e

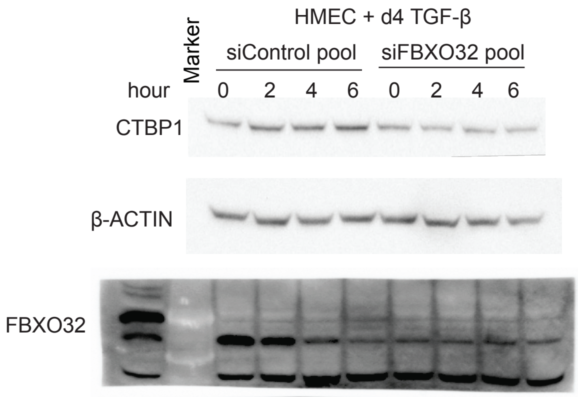

Supplementary Fig. 4i

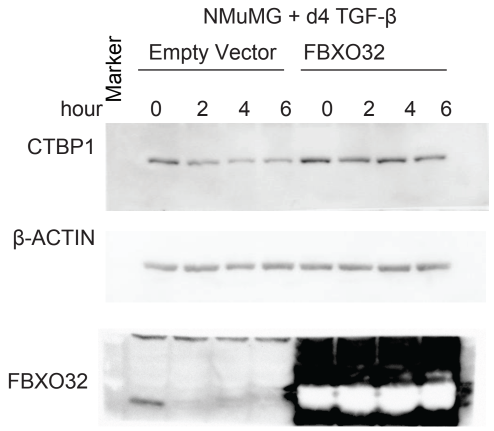

Figure 3g

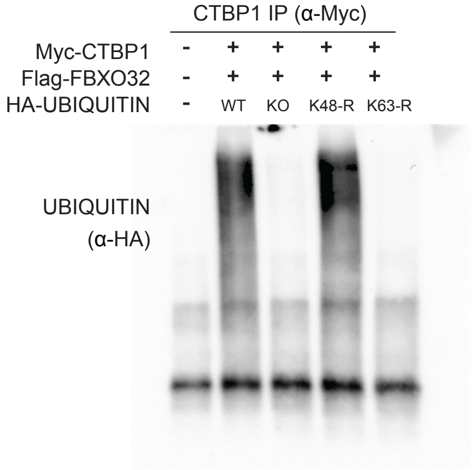

Supplementary Figure 4 e,f

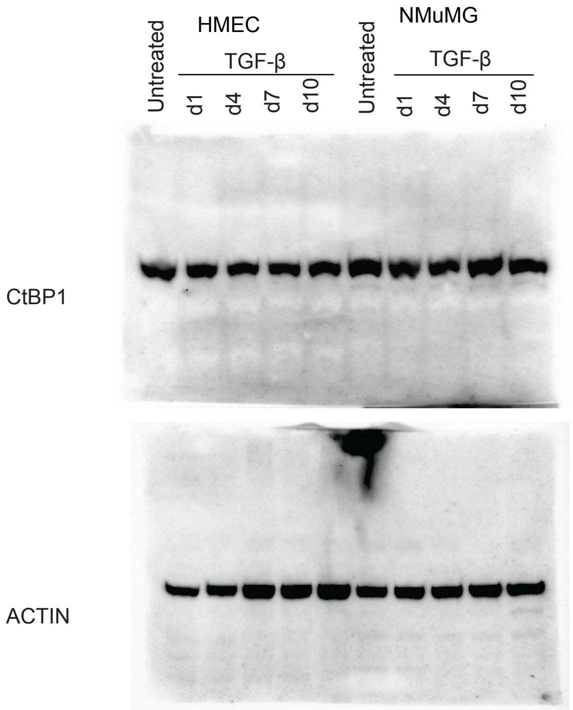

Supplemental Figure 4h

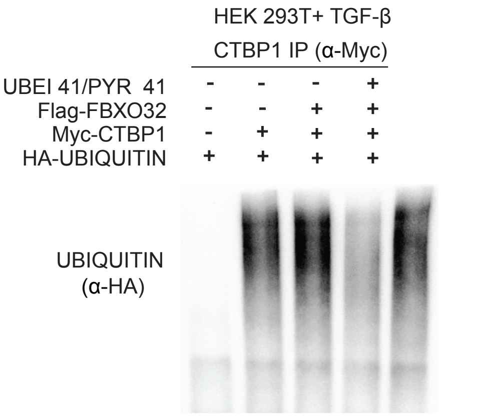

Figure 7b

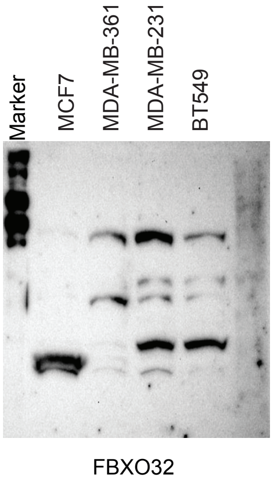

Supplementary Figure 12. Uncropped immunoblot gel images shown in the manuscript. Figure number and antibody used for the blots are indicated.
