# Supplementary material for: FBXO32 promotes microenvironment underlying epithelial-mesenchymal transition via CtBP1 during tumour metastasis and brain development
Source: Nat Commun. 2017 Nov 15;8:1523. doi: 10.1038/s41467-017-01366-x (PMC5688138; doi:10.1038/s41467-017-01366-x)
Supplement: Supplementary file 3 — Description of Additional Supplementary Files [file 41467_2017_1366_MOESM3_ESM.pdf]

Description of Additional Supplementary Files

File name: Supplementary Information
Description: Supplementary figures and legends.

File name: Supplementary Data 1
Description: Table 1 - Primers details used in the manuscript for ChIP and Real-time PCRs. Table 2 - siRNA sequences used in the manuscript for knockdown experiments.

File name: Supplementary Data 2
Description: FBXO32 Immunoprecipitation-mass spectroscopy data.
