# Supplementary material for: FBXO32 promotes microenvironment underlying epithelial-mesenchymal transition via CtBP1 during tumour metastasis and brain development
Source: Nat Commun. 2017 Nov 15;8:1523. doi: 10.1038/s41467-017-01366-x (PMC5688138; doi:10.1038/s41467-017-01366-x)
Supplement: Supplementary file 4 — Supplementary Data 1 [file 41467_2017_1366_MOESM4_ESM.docx]

**Supplementary Data 1**

**Table 1: Mouse RT primers used in the manuscript**

| Ctcf | \| CACACACACAGGTACTCGTCCTCA \| \| --- \| \| CCACTGGTCACAAAGGCCATATC \| |
| --- | --- | --- | --- |
| Tbp | \| GTTTCTGCGGTCGCGTCATTT \| \| --- \| \| TGGGTTATCTTCACACACCATGAA \| |
| Fbxo32 | AGTGAGGACCGGCTACTGTG  GATCAAACGCTTGCGAATCT |
| Cdh1 | \| GAGACAGGCTGGCTGAAAGTGAC  TGACACGGCATGAGAATAGAGGA \| \| --- \| |
| Cdh2 | \| GGTGGAGGAGAAGAAGACCAGGA \| \| --- \| \| TGGCATCAGGCTCCACAGTATCT \| |
| Fn1 | \| CGGAGAGAGTGCCCCTACTA \| \| --- \| \| CGATATTGGTGAATCGCAGA \| |
| Twist1 | \| TCAGCTACGCCTTCTCCGTCTG \| \| --- \| \| TGTCCATTTTCTCCTTCTCTGGAA \| |
| Snai1 | \| TCTCTAGGCCCTGGCTGCTTC \| \| --- \| \| CAGCAAAAGCACGGTTGCAGT \| |
| Zeb2 | \| CCAGAGGAAACAAGGATTTCAGG \| \| --- \| \| CGGAGTCTGTCATGTCATCTAGGC \| |

**Human RT primers used in the manuscript**

| CTCF | \| GGGCTTGAGAGCTGGGTTCTATT \| \| --- \| \| CTTCGACTGCATCACCTTCCATT \| |
| --- | --- | --- | --- |
| TBP | \| GTGAACATCATGGATCAGAACAACA \| \| --- \| \| AAGATAGGGATTCCGGGAGTCAT \| |
| FBXO32 | \| GGTGTATCGGATGGAGACGATTCT \| \| --- \| \| TCAGTGAAGGTGAGGCCTTTGAA \| |
| CLDN3 | \| AACCTGCATGGACTGTGAAACCT \| \| --- \| \| GGTGGTCAAGTATTGGCGGTCAC \| |
| CDH2 | \| GGCCGTCATCACAGTGACAGAT \| \| --- \| \| CCCTGTTCTCAGGAACTTCACCA \| |
| FN1 | \| GCGAGAGTGCCCCTACTACACTG \| \| --- \| \| AATGTTGGTGAATCGCAGGTCA \| |
| TWIST2 | \| GTCCATGTCCGCCTCCCACTA \| \| --- \| \| CAGCATCATTCAGAATCTCCTCCT \| |
| SNAI2 | \| CCTGGTTGCTTCAAGGACACATT \| \| --- \| \| TGTTGCAGTGAGGGCAAGAAAA \| |
| SNAI1 | CAGCGAGCTGCAGGACTCTAATC  AGGATCTCCGGAGGTGGGATG |
| ZEB1 | \| CCAACAGACCAGACAGTGTTACCAG \| \| --- \| \| TCTTGCCCTTCCTTTCCTGTGT \| |
| G0S2 | \| GCAGCACGCCTCCTAGGAACT \| \| --- \| \| GGTCTGTCTCTGTCTACTGCGTCTC \| |
| CXCL3 | \| CCATGGTTCAGAAAATCATCGAAA \| \| --- \| \| TCTGGTAAGGGCAGGGACCAC \| |
| MMP1 | \| CCCCAAAAGCGTGTGACAGTAAG \| \| --- \| \| AAGGGATTTGTGCGCATGTAGAA \| |
| MMP10 | \| TTTCTGCATTTTGGCCCTCTCTT \| \| --- \| \| AACGGTGTCCCTGCTGTTAACTT \| |
| CDH11 | \| CCCTCAGCAAGACCACCGTACA \| \| --- \| \| GTGGTAGGCACAGGAGAATGCAG \| |
| IGFBP2 | \| CTGGAGGAGCCCAAGAAGCTG \| \| --- \| \| AGGGAGTAGAGGTGCTCCAGAGG \| |
| GRAMD2 | \| AATGCAGAGGGGAGGGCTCAT \| \| --- \| \| GGCCCAGAGCATGGAAATTCA \| |
| FGFBP1 | \| GGGAAAAGGAGAACTCAGCACTTT \| \| --- \| \| ACAACACTGTGGCACGTTACTCAC \| |
| BCO2 | \| CAGAATTCACTATTTCCTTGCCACTG \| \| --- \| \| AAACCGAAGCTTGTATGCACTCG \| |
| WNT10A | \| CTGTGGGCTCTAGGACTGACTGG \| \| --- \| \| CAACTGAACTGTCTGGGCTTCG \| |
| NGFR | \| CCTCATCCCTGTCTATTGCTCCA \| \| --- \| \| GTTCTGCTTGCAGCTGTTCCAC \| |
| FAM123B | \| CAGGAATAGGATCCTTACCACCTGA \| \| --- \| \| CTGCTTCTGCAGCCACATATCCT \| |
| IFIT2 | \| GTGGCAGAAGAGGAAGATTTCTGA \| \| --- \| \| TTTTAGTTGCCGTAGGCTGCTCTC \| |
| CCND1 | \| GTGCATCTACACCGACAACTCCAT \| \| --- \| \| ACTTGAGCTTGTTCACCAGGAGCA \| |
| CTBP1 | \| CACGAGTCGGAACCCTTCAGC \| \| --- \| \| CACGAGTCGGAACCCTTCAGC \| |

**Human ChIP Primers used in the manuscript**

| FBXO32 | \| GGCAGTAGCTGCCGCAGTATTTA  GAAAACAAGCCGAGCCCATAAAC \| \| --- \| |
| --- | --- | --- |
| MMP1 | \| CCCCAAAAGCGTGTGACAGTAAG  AAGGGATTTGTGCGCATGTAGAA \| \| --- \| |
| MMP10 | \| TTTCTGCATTTTGGCCCTCTCTT  AACGGTGTCCCTGCTGTTAACTT \| \| --- \| |
| HAS2 | \| CCTCCTCCAACTTAAGGGGGTCT  AGTCCACACCTCCCTCTCCACTT \| \| --- \| |
| CXCR1 | \| CAGATGACACCTCCCTTCTGAGC  TGGACCCTGGCAGTCTCTAATCA \| \| --- \| |
| IGFBP3 | \| TCGTCTACAAGAACCAAGGTGTGC  AGGGAGACCTCACCCCGAGAG \| \| --- \| |
| BMP2 | \| TGGATCCCACGTCTATGCTATGC  AGGGTCAGGGTCTGGCCTCTTAT \| \| --- \| |
| CXCL1 | \| GAGACACAACGCTCTTCCTCCAA  ACTCTGGGATATTCGCCTTCTGC \| \| --- \| |
| INTERGENIC | \| CTACGTTCTCTATGGGGGTGTGC  TGGGTTAAGAATTTGAGGGTAAATGAA \| \| --- \| |

**Table 2:** **Mouse siRNA used in the manuscript**

| Fbxo32 | ACAAGGAGGUAUACAGUAA  UGUUGGAGCUGAUAGCAAA  GCAGAGAGUCGGCAAGUCU  CCUCAAGACUUUAUCAAUU |
| --- | --- |
| Ctbp1 | GCAGCGGGUUUGACAAUAU  AGAAUCAUCGUCCGAAUUG  AUACCUAUCUGAUGGAAUC  CCAUACGAGUGACCAGUUG |

**Human siRNA used in the manuscript**

| FBXO32 | GCAGAUCCGCAAACGAUUA  GUACACUGGUCCAAAGAGU  GUGCUGGUCGGGAACAUUA  CAACUGAACAUCAUGCAGA |
| --- | --- |
| CTBP1 | GGAUAGAGACCACGCCAGU  GAGCAGGCAUCCAUCGAGA  UGAAGAACUGUGUCAACAA  AUGAGAAGGUCCUGAACGA |
